# The Effects of Tinnitus and Tinnitus Annoyance on Need for Recovery After Work: Results of the Netherlands Longitudinal Study on Hearing

**DOI:** 10.1097/AUD.0000000000001323

**Published:** 2022-12-27

**Authors:** Iris A. Simons, Thadé Goderie, Birgit I. Lissenberg-Witte, Niek J. Versfeld, Sophia E. Kramer, Marieke F. van Wier

**Affiliations:** 1 Amsterdam UMC location Vrije Universiteit Amsterdam, Otolaryngology-Head and Neck Surgery, section Ear and Hearing, Amsterdam, The Netherlands; 2 Amsterdam Public Health, Quality of Care, Amsterdam, The Netherlands; 3 Amsterdam UMC location Vrije Universiteit Amsterdam, Epidemiology and Data Science, Amsterdam, The Netherlands; 4 These authors contributed equally to this work and share first authorship.

**Keywords:** Adults, Cross-sectional, Fatigue, Hearing loss, Need for recovery after work, Occupational health, Speech recognition in noise, Tinnitus, Tinnitus annoyance, Workers, Work participation

## Abstract

The first aim of this study was to examine the relationship between having tinnitus and the need for recovery after work (NFR). The second aim was to investigate whether the level of tinnitus annoyance is associated with NFR.

**Design:**

Data from the 5- and 10-year follow-up measurement rounds of the Netherlands Longitudinal Study on Hearing (NL-SH) were used in a cross-sectional analyses. The NL-SH is a web-based prospective cohort study and includes participants aged 18 to 70 years at baseline. For this study, we included only participants who worked at least 12 hours/week and were under the age of 65 years. Participants completed questionnaires on demographic, socioeconomic, psychosocial, hearing-related, and work-related characteristics. In addition, participants answered questions about hearing ability and tinnitus and performed an online digit-triplet speech recognition in noise test to measure the speech reception threshold (SRT) in noise. Participants were asked if (1) they suffer from tinnitus and (2) to rate tinnitus annoyance on a 0-100 numeric rating scale. A linear mixed model was used (1) to estimate the overall (i.e., cross-sectional) association between having tinnitus and NFR and (2) to estimate the overall association between the level of tinnitus annoyance and NFR. The models were checked for effect modification and confounding of factors known to be associated with either tinnitus or NFR and available in the NL-SH.

**Results:**

The study sample comprised 770 unique participants in total; 686 and 335 participants at 5- and 10-year follow-up, respectively. Distress, somatization, and self-reported hearing disability appeared to be confounding factors in the analysis of having tinnitus and NFR. After adjusting for these factors, participants with tinnitus had a 2.5% higher NFR (95% confidence interval: −0.9 to 5.9; *p* = 0.15). In the analysis of tinnitus annoyance and NFR, SRT was an effect modifier. Distress, somatization, depression, and self-reported hearing disability were confounders. After adjustment for effect modification and confounding, tinnitus annoyance was not significantly associated with NFR (*p* = 0.79 for tinnitus annoyance).

**Conclusions:**

This study showed that having tinnitus was not associated with a higher NFR. Also, higher levels of tinnitus annoyance were not associated with a higher NFR. NFR was associated with the psychological factors distress, somatization, and depression, which are known to be intricately related to tinnitus. A longitudinal study design is recommended as it can assess the sequence of events, which might help disentangle the association between tinnitus, NFR, and psychological factors.

## INTRODUCTION

Tinnitus affects millions of people worldwide. The overall prevalence is estimated to range from 5% to 43% (McCormack et al. 2016), with estimations varying because of differences in definition and measurement of tinnitus. The Tinnitus Research Initiative defines tinnitus as “the conscious awareness of a tonal or composite noise for which there is no identifiable corresponding external sound source” (De Ridder et al. 2021). For some people, tinnitus is not, or only mildly annoying, while others experience it as a debilitating condition. Tinnitus can have a negative impact on the quality of life and is associated with psychological factors, primarily anxiety and depression (Nondahl et al. 2007; Trevis et al. 2018). Psychological factors may contribute to the continuous awareness of tinnitus and enhance severity, contributing to a failure to habituate to the sensation of tinnitus (Jastreboff et al. 1996). It can also affect participation in society, such as at work (Kochkin et al. 2011). It was shown that people with tinnitus have longer sick leave spells (Friberg et al. 2013) and a higher likelihood of being granted a disability pension (Friberg et al. 2013; Gustafsson et al. 2011).

Having work is considered an important element of someone’s social environment. It is associated with improved self-reported well-being, reduced depression and anxiety symptoms, a greater sense of autonomy, and promotes mental health (Modini et al. 2016). However, participation in work can also be demanding, feel like a burden, and cause distress and fatigue (Bültmann et al. 2002; Karasek et al. 1998). In a normal recuperation cycle, work-related fatigue is resolved by the next working day (van Veldhoven and Broersen 2003). Tinnitus might affect this recuperation cycle. It can cause difficulties for work performance and affects concentration (Kochkin et al. 2011; Watts et al. 2018). For those reasons, tinnitus might cause more work-related fatigue, and consequently a higher need for recovery after work (NFR) (Degeest et al. 2017; Meikle et al. 2012; Watts et al. 2018). NFR is regarded as “temporary feelings of overload, irritability, social withdrawal, lack of energy for new effort, and reduced performance” (van Veldhoven and Broersen 2003). Elevated levels of NFR are a risk factor for adverse work-related outcomes, such as production losses, sickness absence, and work disability (de Croon et al. 2003; Sluiter et al. 1999, 2003; van Veldhoven and Broersen 2003). A higher NFR might explain for the longer sick leave spells and increased risk for work disability in people with tinnitus. However, it is not clear if tinnitus is indeed associated with a higher NFR.

With this study, we aim to answer the following research questions (RQs): (1) Is having tinnitus associated with a higher NFR compared to people who do not have tinnitus? (2) Is a higher level of tinnitus annoyance associated with a higher NFR compared to people who have no tinnitus?

## METHODS

### Data Collection

The Netherlands Longitudinal Study on Hearing (NL-SH) is an ongoing, web-based prospective cohort study. It includes participants aged 18 to 70 years at study entry. Both normal-hearing and hearing-impaired adults participate. The NL-SH was set up to examine the relationship between hearing ability and different domains of life, such as demographic, socioeconomic, psychosocial, health-related, and work-related characteristics (Goderie et al. 2021; Nachtegaal et al. 2012; Stam et al. 2016). All participants are recruited online. Recruitment, including baseline measurement (T0) at the time of enrollment, started in 2006 and still continues. The second measurement round (T1) started in 2011, and the third measurement round (T2) in 2016. Further details related to the recruitment of participants and follow-up measurements are reported by Stam et al. (2015). For the current study, data collected until February 2021 were used. The NL-SH study, including the follow-up measurement rounds, has been approved by the Medical Ethics Committee of the Amsterdam UMC location Vrije Universiteit Amsterdam, The Netherlands.

### Study Sample

**Fig. 1.**
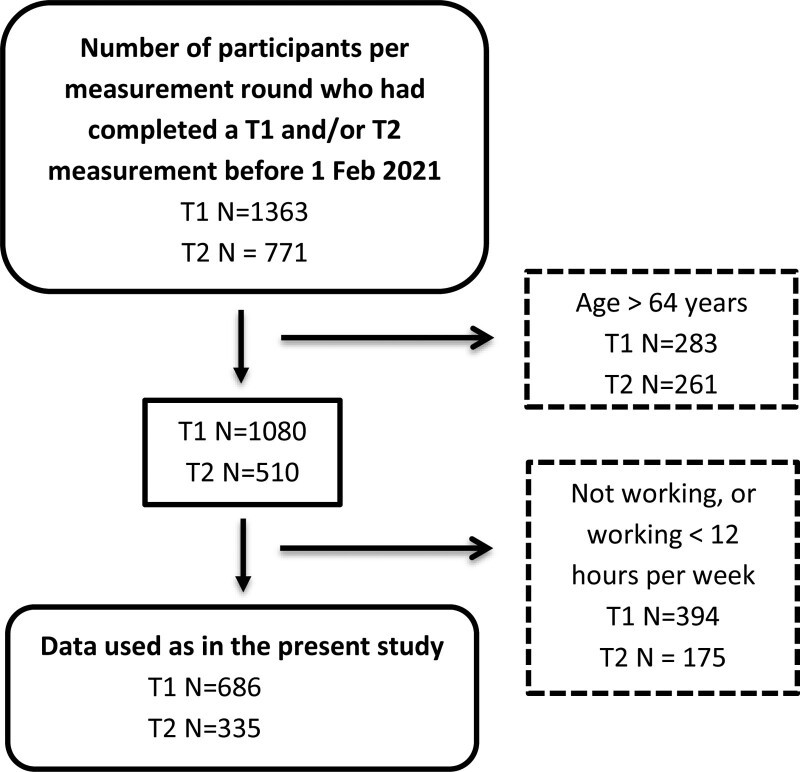
Flow chart of participant numbers after applying inclusion criteria per measurement round. The number of measurements excluded per measurement round are given in the dashed line rectangle. T1 = first included measurement, T2 = second included measurement and 5 years later than T1.

For this study, we used data from measurement rounds T1 and T2 (Fig. 1), as tinnitus annoyance was not measured in round T0. We included participants who had a paid job for 12 hours or more per week and were in the age range of 18 to 64 years. The 12-hour threshold was chosen based on the definition of Statistics Netherlands (a Dutch governmental institution that gathers statistical information about the Netherlands), which defines the labor force as working 12 hours/week or more (StatisticsNetherlands 2015). The age of 18 to 64 years was considered the working age population, as the average retirement age in the Netherlands was just under 65 years at the time the data were collected (StatisticsNetherlands 2019).

### Dependent Variable

#### Need for Recovery After Work

The NFR questionnaire is a reliable and valid instrument assessing the short-term effects of fatigue caused by work activities (e.g., “I find it difficult to concentrate in my free time after work”) (van Veldhoven and Broersen 2003). It is an 11-item questionnaire in which each item has two response options: “yes” or “no.” The number of items answered with “yes” was divided by the total number of items and multiplied by 100 to obtain a percentage score. Higher scores indicate a higher NFR. A cutoff score of 55% has a 72% sensitivity and a 79% specificity for identifying people with increased risk for psychological symptoms (Broersen et al. 2004).

### Independent Variables

#### Tinnitus

To assess the presence of tinnitus, participants were asked: “Do you suffer from ringing in the ears (tinnitus)?” Response options were “yes,” “no,” or “I don’t know.” Participants answering “yes” were considered having tinnitus. Those answering “no” or “I don’t know” were considered having no tinnitus. Participants who responded “yes” were asked to rate their typical tinnitus annoyance using a numeric rating scale (NRS). They were asked “How annoyed are you by your tinnitus in everyday life on an NRS from 0 to 100, with 0 = no annoyance at all and 100 = extremely annoying” in accordance with Meikle et al. (2008) and Kim et al. (2016). Because of nonlinearity, tinnitus annoyance scores were categorized into six groups, that is: no tinnitus, NRS = 0 to 20, NRS = 21 to 40, NRS = 41 to 60, NRS = 61 to 80, and NRS = 81 to 100. The group with tinnitus annoyance scores in the 81–100 category was too small (N < 10) for analysis so it was merged with the group with an NRS of 61–80.

### Potential Confounders and Effect Modifiers

Factors available in the NL-SH database which were possibly associated with either tinnitus or NFR were analyzed as potential confounders or effect modifiers. These included the demographic, socioeconomic and lifestyle variables age, sex, tobacco smoking, educational level, and working hours per week (Unterrainer et al. 2001; Jansen et al. 2002; Fujii et al. 2011; Kim et al. 2015; Veile et al. 2018). Hearing-related factors were speech recognition in noise, hearing aid use, self-reported hearing disability, occupational noise exposure, and hyperacusis (Baguley et al. 2013; Nachtegaal et al. 2009; Tyler et al. 2014; Van Leeuwen et al. 2021). The work-related factor was social support at work (Karasek et al. 1998). Psychological factors were distress, anxiety, depression, and somatization (Jansen et al. 2002; Trevis et al. 2018).

- Demographic and lifestyle characteristics assessed were age (range 18–64 years), sex (male/female), tobacco smoking status, and educational level. Smoking was categorized into “current smoker,” “history of smoking,” and “never smoked.” The educational level was divided into three groups: “low” (elementary school or attended high school but no degree), “mid” (high school graduate or having an associate degree), and “high” (having a bachelor’s, master’s, or doctoral degree).
- *Working hours*. Participants were considered “working” if they had a paid job or were a business owner and worked at least 12 hours/week. These participants were asked how many hours they worked per week.
- *Occupational noise exposure*. Occupational noise exposure was evaluated using the question: “How often are you exposed to loud sounds or noises at work?.” The item comprises four categories: “almost never,” “occasionally,” “frequently,” and “almost always.”
- *Speech recognition in noise.* Speech recognition in noise was measured using the national hearing test. This is an adaptive online screening test that measures the ability to correctly repeat digit triplets in background noise (e.g., 6-2-5) by determining the speech reception threshold in noise (SRT) and is expressed as a signal-to-noise ratio (in dB SNR) (Smits et al. 2006b). Participants are instructed to do the test in a quiet room. Hearing aid users are instructed to do the test without their hearing aid(s). Participants are asked to indicate the type of transducer they use (i.e., headphones or speakers), but are advised to use headphones. The test is binaural and measures the best ear. A total of 23 digit triplets are presented against a stationary background noise; after a correct response, the next triplet is presented at a 2-dB lower SNR, whereas the opposite happens after an incorrect response. The SRT was calculated by taking the average SNR of the last 20 triplets, which corresponds to 50% speech intelligibility (Smits et al. 2006b). Lower SRT-scores signify better speech recognition in noise and higher SRT-scores worse speech recognition in noise. For descriptive purposes, the National Hearing Test scores were categorized as good (SRT < −5.5 dB SNR), insufficient (−5.5 dB SNR ≤ SRT ≤ −2.8 dB SNR), or poor (SRT > −2.8 dB SNR) (Smits et al. 2006a).
- *Hearing aids* or *cochlear implants.* The use of hearing aids and cochlear implants was derived from the question “do you use hearing aids” or “do you use a cochlear implant?” Answer categories were “yes” or “no.”
- *Self-reported hearing disability*. Self-reported hearing disability in daily life was determined using the Amsterdam Inventory for Auditory Disability and Handicap (AIADH) (Boeschen Hospers et al. 2016; Kramer et al. 1995). It comprises 28 questions. An item example is “can you carry on a conversation with someone during a crowded meeting?.” Response options were “almost always” (0), “frequently” (1), “occasionally” (2), “almost never” (3). The sum scores range from 0 to 84, with higher scores indicating increased subjective hearing disability.
- *Hyperacusis*. Hyperacusis was assessed using the item: “Do you feel that loud noises bother you more than they bother other people (hyperacusis)?.” Answer categories were “yes,” “no,” or “I don’t know.” Participants answering “yes” were considered having hyperacusis. Those answering “no” or “I don’t know” were considered having no hyperacusis.
- *Social support at work.* Social support at work was measured using 8 items from the “Job Content Questionnaire” (Karasek et al. 1998). An item example is: “People I work with take a personal interest in me.” Answers were given on a 4-point scale, varying from strongly disagree (scored as 1) to strongly agree (scored as 4). Scores range from 8 to 32, with higher scores indicating better social support at work.
- *Psychological symptoms.* Psychological symptoms concerned distress, depression, anxiety, and somatization. These were assessed using the “Four-dimensional symptom questionnaire,” a multidimensional self-report questionnaire (4DSQ) (Terluin et al. 2006). Response categories on all four scales are divided into “no” (0), “sometimes” (1), and “regularly” or “often or constantly” (2). Sum scores were calculated. The anxiety scale comprised 12 items (score range 0–24). An example item is: “During the past week, did you have any fear of going out of the house alone?” The depression scale comprised 6 items (score range 0-12). An example item is: “During the past week, did you feel everything is meaningless?” The somatization scale comprised 16 items (score range 0-32). An example item is: “During the past week, did you suffer from tingling in the fingers?” The distress scale comprised 16 items (score range 0-32). An example item is: “During the past week, did you feel tense?” Higher scores indicate worse psychological symptoms.

### Statistical Analysis

Means and standard deviations (SD) were calculated for the normally distributed continuous variables. Medians and interquartile ranges (IQRs) were calculated for non-normally distributed continuous variables. Percentages were calculated for categorical variables. For both RQs, a linear mixed model was used, because it can adjust for multiple measurements within participants. In RQ1, NFR was the dependent variable and having tinnitus the independent variable. In RQ2, NFR was the dependent variable and tinnitus annoyance the independent variable. Tinnitus annoyance was categorized with having no tinnitus as a reference group. For both RQs, we used the same procedure. First, the model was checked for effect modification by adding an interaction term to the model (potential effect modifier * tinnitus), with *p* < 0.05 indicating effect modification. In case of effect modification with a categorical variable, a stratified analysis was performed. In case of effect modification with a continuous variable, the interaction was graphically illustrated per category and the interaction was kept in the regression model. Second, the model was checked for confounding. A variable was considered a confounder and included in the model if:

1. The potential confounder was significantly associated with the dependent variable and the independent variable (*p* < 0.05). In the case of a continuous potential confounder, linear mixed model analysis was used to evaluate the association between the potential confounder and the dependent variable (NFR), and between the potential confounder and the independent variable (tinnitus or tinnitus annoyance). In case a potential confounder was a categorical variable, generalized estimating equations were used.
2. The regression coefficient of having tinnitus (RQ1) or tinnitus annoyance (RQ2) changed ≥ 10% after adding the potential confounder to the model.

Variables satisfying the criteria for confounding were added one by one in a forward stepwise procedure. The linearity assumptions for linear mixed model analysis were verified. In case of nonlinearity, continuous variables were categorized. Multicollinearity was evaluated in the final model to assess the correlation between one or more of the independent variables. If high variance inflation factor (VIF) values were observed, the clinically least relevant variable was removed from the model. The analysis was cross-sectional in nature as the dependent variable and the independent variables were measured at the same moment (i.e., at T1 and/or T2). The significance level was set at 0.05 for all statistical analyses. Analyses were performed using SPSS version 26.0 (IBM Corp., Armonk, NY, USA).

## RESULTS

Complete data on speech recognition in noise and tinnitus outcomes were available for N = 1363 at T1 and N = 771 participants at T2. After applying exclusion criteria (Fig. 1), at T1 N = 686 and at T2 N = 335 participants remained. The total sample contained N = 770 unique participants, of which N = 251 participants completed the measurements at both T1 and T2. Table 1 summarizes the descriptive statistics for the included participants at both time points (T1 and T2). The tinnitus prevalence at T1 was 37.6% (95% confidence interval [CI], 34.0–41.2) and at T2 42.4% (95% CI, 37.1–47.7). At T1, 9/251 participants (3.6%) reported tinnitus who did not report this at T2. At T2, 24 of 251 participants (9.6%) reported tinnitus, who had not reported this at T1. The sample included more women than men. Over half of the participants reported a high level of education.

**TABLE 1.** Participant characteristics for each measurement round.

	T1		T2	
	No Tinnitus, N= 428	Tinnitus, N=258	No Tinnitus, N= 193	Tinnitus, N=142
Age, mean (SD), y	46.2 (11.1)	50.3 (10.0)	48.5 (9.5)	51.3 (8.7)
Range, y	24 to 64	23 to 64	29 to 64	31 to 64
Sex				
Female, n (%)	306 (71.5)	159 (61.6)	138 (71.5)	96 (67.6)
Male, n (%)	122 (28.5)	99 (38.4)	55 (28.5)	46 (32.4)
Tobacco smoking*				
Never, n (%)	208 (48.6)	115 (44.6)	112 (58.0)	63 (44.4)
Former, n (%)	159 (37.1)	110 (42.6)	59 (30.6)	60 (42.3)
Current, n (%)	61 (14.3)	32 (12.4)	22 (11.4)	19 (13.4)
Missing, n (%)	—	1 (0.4)	—	—
Educational level				
Low, n (%)	35 (8.2)	26 (10.1)	7 (3.6)	11 (7.7)
Mid, n (%)	103 (24.1)	75 (29.1)	52 (26.9)	43 (30.3)
High, n (%)	290 (67.8)	157 (60.9)	134 (69.4)	88 (62.0)
Speech recognition in noise (SRT)				
Mean (SD), dB SNR	−5.1 (3.5)	−3.8 (4.1)	−5.1 (3.5)	−4.2 (4.3)
Range, dB SNR	−10.0 to 4.0	−13.4 to 4.0	−10.2 to 4.0	−10.0 to 4.0
Good, n (%)	262 (61.2)	109 (42.2)	115 (59.6)	81 (57.0)
Insufficient, n (%)	76 (17.8)	59 (22.9)	38 (19.7)	12 (8.5)
Poor, n (%)	90 (21.0)	90 (34.9)	40 (20.7)	49 (34.5)
AIADH, median (IQR), score	12 (3.0–30.8)	29 (10.8–44.3)	11 (3.0–29.0)	26 (8.8–43.3)
Range	0 to 84	0 to 80	0 to 63	0 to 74
Hearing aid				
Yes, n (%)	102 (23.8)	111 (43.0)	51 (26.4)	65 (45.8)
No, n (%)	326 (76.2)	147 (57.0)	142 (73.6)	77 (54.2)
Cochlear implant				
Yes, n (%)	6 (1.4)	9 (3.5)	4 (2.1)	7 (4.9)
No, n (%)	422 (98.6)	249 (96.5)	189 (97.9)	135 (95.1)
Tinnitus annoyance, mean (SD)	—	38.6 (26.1)	—	37.0 (26.7)
Score 0–20, n (%)	—	90 (34.9)	—	56 (39.4)
Score 21–40, n (%)	—	59 (22.9)	—	34 (23.9)
Score 41–60, n (%)	—	51 (19.8)	—	23 (16.2)
Score 61–80, n (%)	—	49 (19.0)	—	20 (14.1)
Score 81–100, n (%)	—	9 (3.5)	—	9 (6.3)
Hyperacusis				
Yes, n (%)	105 (24.5)	112 (43.4)	59 (30.6)	67 (47.2)
No, n (%)	323 (75.5)	146 (56.6)	134 (69.4)	75 (52.8)
Working hours/week, Mean (SD), hours	34.1 (11.0)	33.7 (11.2)	32.9 (11.7)	33.2 (10.4)
Range, hrs	12–70	12–70	12–70	12–75
NFR, median (IQR), range 0–100	36 (9–64)	55 (18–82)	36 (9–64)	55 (18–82)
NFR > 54*, n (%)	165 (38.6)	133 (51.6)	81 (42.0)	81 (57.0)
Distress, median (IQR), range 0–32	5 (2.0–10.0)	7 (3.0–14.0)	5 (2.0–11.0)	8 (3.8–12.3)
Somatization, median (IQR), range 0–32	4 (2.0–8.0)	6 (3.0–10.0)	4 (2.0–8.0)	6 (3.0–11.0)
Anxiety, median (IQR), range 0–24	0 (0.0–1.0)	0 (0.0–2.3)	0 (0.0–2.0)	1 (0.0–2.0)
Depression, median (IQR), range 0-12	0 (0.0–1.0)	0 (0.0–1.0)	0 (0.0–0.0)	0 (0.0–1.0)

* *Scores >54 on the NFR scale indicate an increased risk of developing psychological symptoms.*

AIADH indicates Amsterdam Inventory for Auditory Disability and Handicap; IQR, interquartile range; N, number; NFR, need for recovery; SD, standard deviation; SRT, speech reception threshold; SNR, signal-to-noise ratio; T1, first included measurement; T2, second included measurement 5 years after T1.

published online ahead of print December 27, 2022.

### Association Between Having Tinnitus and NFR (RQ1)

The crude model showed that participants reporting to have tinnitus had a 9.8% (95% CI, 5.8–13.9; *p* < 0.001) higher NFR compared to nontinnitus participants (Table 2). No effect modification was found. Distress, somatization, and the AIADH score were identified as confounders. The model showed moderate collinearity with VIF values of 1.5, 1.5, and 1.1, for each of these confounders, respectively. This indicates some redundancy in the regression model but does not necessitate changing the model. After correcting for confounders, participants with tinnitus had a statistically non-significant 2.5% (95% CI, −0.9 to 5.9; *p* = 0.15) higher NFR compared to participants without tinnitus (Table 2).

**TABLE 2.** Crude and adjusted models of the association between having tinnitus and the NFR

	β	95% CI (for β)	*p*
Crude model			
No tinnitus	0		
Tinnitus	9.8	5.8 to 13.9	<0.001
Adjusted model*			
No tinnitus	0		
Tinnitus	2.5	−0.9 to 5.9	0.15
Distress	2.2	1.9 to 2.5	<0.001
Somatization	0.8	0.4 to 1.2	<0.001
AIADH	0.21	0.12 to 0.30	<0.001

The effect size is the regression coefficient in NFR percentage. β is the regression coefficient of the independent variable.

* Adjusted model is corrected for the confounders distress, somatization, and AIADH.

AIADH indicates Amsterdam Inventory for Auditory Disability and Handicap (self-reported hearing disability score); CI, confidence interval; NFR, need for recovery.

### Association Between Tinnitus Annoyance and NFR (RQ2)

**Fig. 2.**
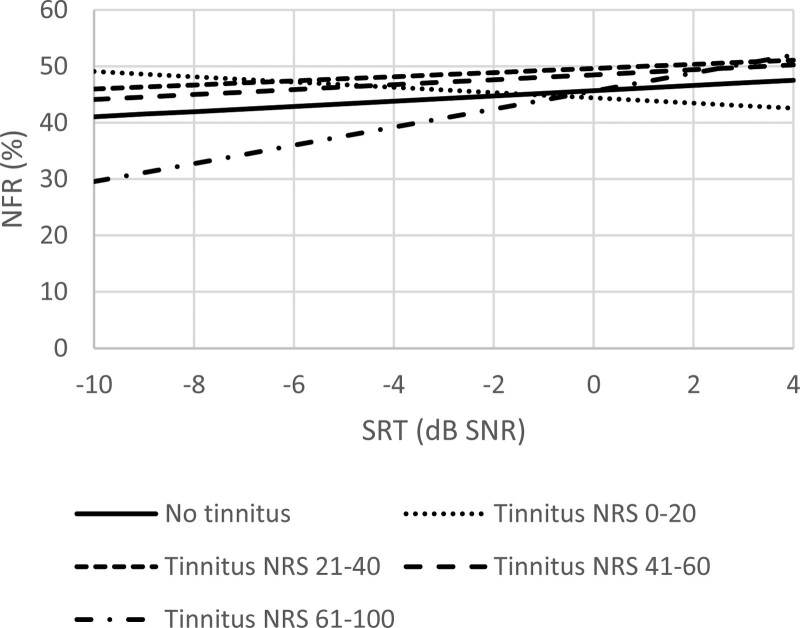
Estimated mean NFR per tinnitus annoyance category. A higher tinnitus NRS indicates a higher tinnitus annoyance. Model adjusted for the mean distress, mean somatization, mean depression, and mean self-reported hearing disability. NFR indicates need for recovery; NRS, numeric rating scale; SNR, signal-to-noise ratio; SRT, speech reception threshold in noise.

In the crude model, tinnitus annoyance was positively associated with NFR (*p* < 0.001; Table 3). SRT was an effect modifier. Distress, somatization, depression, and AIADH confounded the association. The model indicated moderate collinearity with VIF values of 2.7, 1.6, 2.1, and 1.2, for each of these confounders, respectively. After adjusting for these confounders, tinnitus annoyance was not associated with NFR (*p =* 0.79) and the interaction with SRT became not significant (*p =* 0.20) (Table 3). The estimated NFR per annoyance category has been visualized in Figure 2.

**TABLE 3.** Crude and adjusted model of the association between tinnitus annoyance and the NFR

	β	95% CI (for β)	*p*
Crude model			
No tinnitus	0		<0.001
Tinnitus annoyance 0–20	7.2	1.9 to 12.5	
Tinnitus annoyance 21–40	10.8	4.2 to 17.3	
Tinnitus annoyance 41–60	10.9	3.8 to 18.0	
Tinnitus annoyance 61–100	13.5	6.4 to 20.6	
Adjusted model*			
No tinnitus	0		0.79
Tinnitus annoyance 0–20	−1.2	−9.0 to 6.5	
Tinnitus annoyance 20–40	4.0	−4.2 to 12.1	
Tinnitus annoyance 40–60	2.8	−4.7 to 10.4	
Tinnitus annoyance 60–100	−0.1	−7.7 to 7.6	
Distress	2.4	2.1 to 2.8	<0.001
Somatization	0.8	0.5 to 1.2	<0.001
Depression	−1.4	−2.7 to −0.1	0.038
AIADH	0.17	0.05 to 0.29	0.007
Speech recognition in noise (SRT)	0.46	−0.25 to 1.20	0.13
Tinnitus annoyance† SRT			0.20
Tinnitus annoyance 0–20† SRT	−0.9	−2.2 to 0.3	
Tinnitus annoyance 20–40† SRT	−0.1	−1.5 to 1.3	
Tinnitus annoyance 40–60† SRT	0.0	−1.3 to 1.3	
Tinnitus annoyance 60–100† SRT	1.1	−0.3 to 2.5	

The effect size is the regression coefficient in NFR percentage. β is the regression coefficient of the independent variable.

* Adjusted Model is corrected for the effect modifier speech reception threshold and the confounders distress, somatization, and AIADH.

† An interaction with effect modifier SRT.

AIADH indicates Amsterdam Inventory for Auditory Disability and Handicap (self-reported hearing disability score); CI, confidence interval; NFR, need for recovery; SRT, speech reception threshold.

## DISCUSSION

After adjustment for the confounders distress, somatization, and self-reported hearing disability (AIADH), tinnitus sufferers did not significantly differ in NFR compared to people without tinnitus. Also, after adjustment for the interaction with SRT and the confounders distress, somatization, depression, and self-reported hearing disability, no association was found between different levels of tinnitus annoyance and NFR compared to having no tinnitus.

### Comparison With Other Studies

Juul Jensen et al. (2018) found a significant association between having tinnitus and a higher NFR. They also found a moderate correlation between the tinnitus handicap index and NFR, with a higher tinnitus handicap index associated with a higher NFR. However, their study population was small and quite restricted, with 32 hearing aid users of whom 16 had chronic tinnitus. Effect modification and potential confounding were not considered. Like us, Van der Hoek-Snieders et al. (2020) also did not find an association between tinnitus and NFR, but they did not take psychological factors into account. Psychological factors should be considered, as they are both associated with tinnitus and NFR (Jansen et al. 2002; Trevis et al. 2018). In contrast to our study, both studies included only hearing-impaired participants. This does not represent the entire group of tinnitus sufferers, as not all of them experience hearing loss (Nondahl et al. 2002; Shargorodsky et al. 2010). Additionally, the level of hearing ability should be considered as a potential confounder, as it affects both NFR and tinnitus (Nachtegaal et al. 2009; Shargorodsky et al. 2010; Nondahl et al. 2011; Van Leeuwen et al. 2021; Goderie et al. 2022). Nachtegaal et al. (2009) found that employees with a reduced hearing ability have a higher NFR. Van Leeuwen et al. (2021) longitudinally analyzed this association and found that a decrease in hearing ability was associated with an increase in NFR. SRT was not a confounder in RQ1, but self-reported hearing disability was. In RQ2, an interaction was seen between SRT and tinnitus annoyance, and self-reported hearing disability was a confounder.

### Influence of Psychological Factors on NFR and Tinnitus

The association between tinnitus, NFR, and psychological factors is complex. Psychological symptoms are associated with NFR and are intricately related to tinnitus in many people suffering from tinnitus (Jansen et al. 2002; Pinto et al. 2014; Van Venrooij et al. 2015; Trevis et al. 2018). When asking if someone has tinnitus, no distinction is made between tinnitus and the psychological reactions to tinnitus. De Ridder et al. (2021) therefore propose to distinguish between “tinnitus” and “tinnitus disorder.” They define “tinnitus” as the “conscious awareness of a tonal or composite noise for which there is no identifiable corresponding external acoustic source, which becomes a ‘tinnitus disorder’ when associated with emotional distress, cognitive dysfunction, and/or autonomic arousal, leading to behavioral changes and functional disability” (De Ridder et al. 2021). In our analysis on the association between tinnitus and NFR (RQ 1), we adjusted for the confounding effect of the psychological factors distress and somatization. After adjusting the crude model for the confounding effects of distress and somatization, no association was found between tinnitus and NFR. The psychological factors distress and somatization could be part of “tinnitus disorder” in some participants. By including these factors in the model, we could over-adjust for these factors and obscure a possible association between tinnitus and NFR. However, only moderate multicollinearity was found between these factors and tinnitus, which disputes a correlation and over-adjustment. It would however be insightful to study the relation between tinnitus and these psychological factors, and the direction of the relation. It might be that tinnitus causes a higher NFR, distress, and somatization. Alternatively, distress and somatization could cause a higher NFR, and tinnitus is not part of the causal pathway in developing an increased NFR. A longitudinal study analyzing change in NFR in people who develop tinnitus could answer this question. Difficulties in such a study are that it assumes a lag time between developing tinnitus and suffering the consequences of tinnitus. If such a lag time is relatively short, such a longitudinal study would need relatively short measurement intervals which can be costly and is demanding for participants in the study.

The problem of fatigue after work is highly prevalent (Bültmann et al. 2002). This research provides insight into the relation between tinnitus, psychological problems, and NFR. The univariable association between tinnitus and need for recovery indicates that general practitioners and occupational physicians should be aware that, in people who report tinnitus, elevated levels of NFR might be observed. Furthermore, they should be aware that the relation between tinnitus and NFR is confounded by psychological factors like distress and somatization. The psychological factors involved in tinnitus should be discussed by health care professionals who treat people with tinnitus.

### Strengths and Limitations

The strengths of the study are that it includes a relatively large sample of participants active in the labor force. A website was used to recruit and enroll participants, creating a heterogeneous group with normal-hearing and hearing-impaired participants. Because of that, tinnitus annoyance levels might be more representative of those in the general population, as opposed to studies in patients who visit a physician because of their tinnitus and who typically have more severe complaints. Both hearing ability, tested with a speech recognition in noise test, and self-reported hearing disability were considered. The questionnaire contained details about work, psychological characteristics, and hearing. Since tinnitus, psychological symptoms, and hearing disability often co-exist, detailed information on these variables is important to study the relationship. Also, participants had wide-ranging scores on the NFR scale.

The study also has limitations. First, we only took the factors into account that were available from the NL-SH. The development of tinnitus, and tinnitus becoming chronic, is a multifactorial process, and the same applies to an increased NFR. The possibility exists that unidentified factors important to that process were not included in the factors available from the NL-SH, despite the extensive coverage of potential confounders and effect modifiers. Second, the question measuring tinnitus was: “Do you suffer from ringing in the ears (tinnitus)” with a yes/no response. This question does not involve temporal characteristics of tinnitus, such as the duration of a tinnitus episode, or the frequency of occurrence in case of episodic tinnitus. Brief transient episodes of tinnitus may have been included in the tinnitus group, but if these brief episodes are infrequent, they are unlikely to affect NFR. Asking about duration would have helped to differentiate chronic tinnitus (i.e., tinnitus with a duration of at least 3 months) from acute tinnitus (De Ridder et al. 2021).

## CONCLUSIONS

This study showed that having tinnitus was not associated with a higher NFR. Also, higher levels of tinnitus annoyance are not associated with a higher NFR. NFR was associated with the psychological factors distress, somatization, and depression, which are known to be intricately related to tinnitus. For future research, a longitudinal study design is recommended, as it can assess NFR before developing tinnitus. This way the sequence of events can be established regarding the development of tinnitus, the change in NFR, and the change in the level of psychological dysfunction.

## ACKNOWLEDGEMENTS

We are grateful to the participants of the Netherlands Longitudinal Study on Hearing (NLSH), without them the research would not have been possible.
